# A Step-by-Step Description of Immediate Loading Provisionalization Following Computer-Guided Surgery and Provisional Printed Prosthesis Placement: A Case Report

**DOI:** 10.7759/cureus.90575

**Published:** 2025-08-20

**Authors:** Maycol Ugaz Urviola, Sergio Charifker Ribeiro Martins, Leandro Lécio de Lima Sousa, Gustavo Fernandes, Tárcio H Ishimine Skiba

**Affiliations:** 1 Periodontics, SOEP - Pós-Graduações, Porto Velho, BRA; 2 Department of Periodontology, Guarulhos University, Guarulhos, BRA; 3 Periodontics, A. T. Still University - Missouri School of Dentistry and Oral Health, St. Louis, USA; 4 Implantology and Oral Rehabilitation, SOEP, Porto Velho, BRA

**Keywords:** all-on-four, angled implants, dental prosthesis, guided surgery, immediate loading, stackable guide

## Abstract

Dental implants have revolutionized the scope of oral rehabilitation in patients requiring tooth replacement. Immediate loading procedures for edentulous patients have become increasingly popular among both clinicians and patients. This report aimed to describe the installation of four flapless-placed implants through prototyped guided surgery and the immediate loading of a printed and prototyped temporary prosthesis. The report presents the digital planning and execution of a full-arch implant rehabilitation in a 49-year-old edentulous male patient with congenital tooth absence. Through the integration of cone beam CT (CBCT) imaging, intraoral scanning, and specific software, a virtual treatment plan was developed to guide implant placement. A custom three-part stackable surgical guide and a stereolithographically printed provisional prosthesis were fabricated to ensure precision. Guided implant surgery was performed under local anesthesia, with all implants achieving primary stability (>32 N·cm), allowing for immediate loading. The provisional prosthesis was relined and secured on titanium abutments. The procedure was completed efficiently within two hours and 10 minutes, demonstrating the effectiveness of a fully digital workflow in complex oral rehabilitation cases. The use of provisional prototypes and guided surgery can yield satisfactory and immediate results for these patients, bringing comfort and practicality to this dental procedure.

## Introduction

Dental implants have demonstrated a high level of survival rate, enabling feasible and predictable oral rehabilitation [1,2]. Several types of rehabilitation options for implants have been proposed, including fixed single crowns, fixed bridges, overdentures, and fixed total dentures [3,4]. One of the most common techniques employed for fixed total rehabilitation is the All-on-4^®^ technique [5]. It is a system that allows fixed complete rehabilitation on dental implants in edentulous patients’ upper and lower arches. This technique provides the patient with a fixed and immediate rehabilitation, avoiding transition periods with removable provisional prostheses [6,7].

Currently, cone-beam CT (CBCT), along with specialized software, has enabled virtual surgical and prosthetic planning based on accurate three-dimensional (3D) bone visualization and its relationship with the future prosthesis [8]. When conducting virtual planning, it is possible to assess and measure bone density; select type, size, and number of implants; determine its location, inclination, and depth level with respect to the bone; analyze the characteristics of the intermediate abutments and emergence of the fixation screw of the future prosthesis; and check the correct positioning of rings in the surgical guide and the ideal location of fixation pins, which are responsible for the stability of the guide in the mouth during surgery [9,10]. Guided surgery planning enables the use of advanced techniques, such as minimally invasive flapless surgeries (flapless or with a flap) [11,12], leading to less trauma, reduced surgery duration, improved postoperative outcomes, and the absence of sutures, while maintaining the integrity of peri-implant tissues [13].

Immediate implant placement and loading [14-16] offer the advantage of reducing total treatment time, preserving alveolar bone, and enhancing patient satisfaction by providing functional rehabilitation shortly after surgery. In the mandible, immediate loading is particularly advantageous due to higher primary stability achievable in dense mandibular bone compared to the maxilla. Furthermore, immediate protocols may improve patient adaptation and minimize the period of functional and aesthetic compromise associated with delayed loading approaches. Immediate loading procedures [14-16] have become increasingly popular among both clinicians and patients. High survival rates and a low incidence of complications demonstrate the predictability of implant treatment, regardless of the loading regimen involved. The challenge today is not to prove functionality but rather to develop cost-effective and straightforward protocols [7,17]. Full-arch mandibular rehabilitation presents specific clinical challenges due to the anatomical constraints of the edentulous mandible, including reduced bone height in the posterior region, higher bone density, and proximity to the inferior alveolar nerve. These factors necessitate careful implant positioning and prosthetic planning to ensure long-term stability and patient comfort.

This case report aimed to present a clinical scenario involving osteotomy, guided and immediate implant placement, and the installation of a 3D-printing resin hybrid prosthesis [18]. All the procedures were planned and carried out in a guided computerized setting. The implants used in this study featured a conical connection and surface modifications designed to enhance osseointegration and biomechanical stability. The macro-design facilitates primary stability in dense mandibular bone, while the platform-switching concept helps preserve crestal bone levels. These design elements, combined with precise surgical and prosthetic execution, contribute to the predictable success of immediate loading protocols in full-arch mandibular rehabilitation. The novelty in this case involved the use of digital workflow, a custom three-part stackable surgical guide, and a stereolithographically printed provisional prosthesis made from biocompatible resin, combined with immediate loading.

## Case presentation

**Figure 1:**
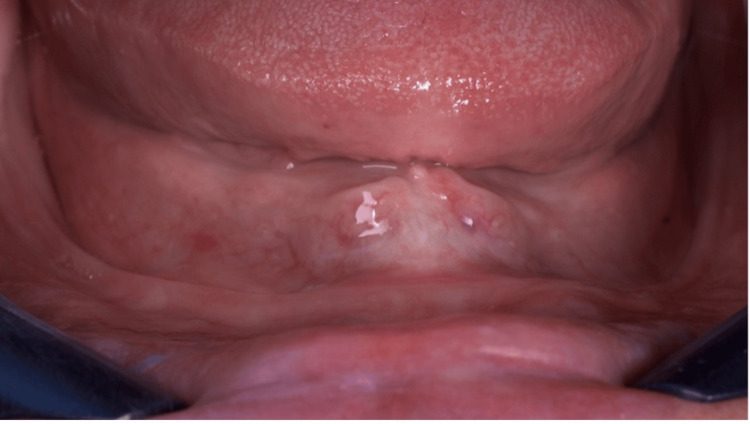
Initial frontal picture of the mandibular arch

A 49-year-old male Caucasian patient presented to our clinic with complaints of masticatory dysfunction and the absence of teeth since childhood. During the initial consultation, a thorough anamnesis, including medical and dental history, was obtained, followed by a clinical dental examination and standardized photographic documentation (Figure 1). No local or systemic contraindications to oral surgery were identified. The patient had a maxillary and mandibular total denture for over 28 years.

The following treatment options were presented to the patient: (1) All-on-4^®^: four implants placed in the mandible to support a fixed-screwed rehabilitation; (2) rehabilitation with total dentures; or (3) overdentures. These options offer possibilities tailored to address functional, esthetic, and psychological needs. Overdentures, specifically, offer a promising alternative for completely edentulous patients, leveraging osseointegrated implants to enhance stability and support. The advantages of using overdentures include lower costs, maintenance of labial support, and reduced invasiveness compared to fixed-screwed prostheses on implants, making them particularly attractive to patients reluctant to embrace total edentulousness due to esthetic concerns. Additionally, overdentures significantly improve oral health-related quality of life (OHRQoL), evidenced by higher satisfaction rates and improved masticatory function when compared to traditional complete dentures. However, while their advantages are notable, challenges regarding oral hygiene maintenance and potential bulkiness persist, necessitating careful management to avoid secondary complications like peri-implant disease. Therefore, since the patient was already using a complete denture, a new one was immediately rejected.

**Figure 2:**
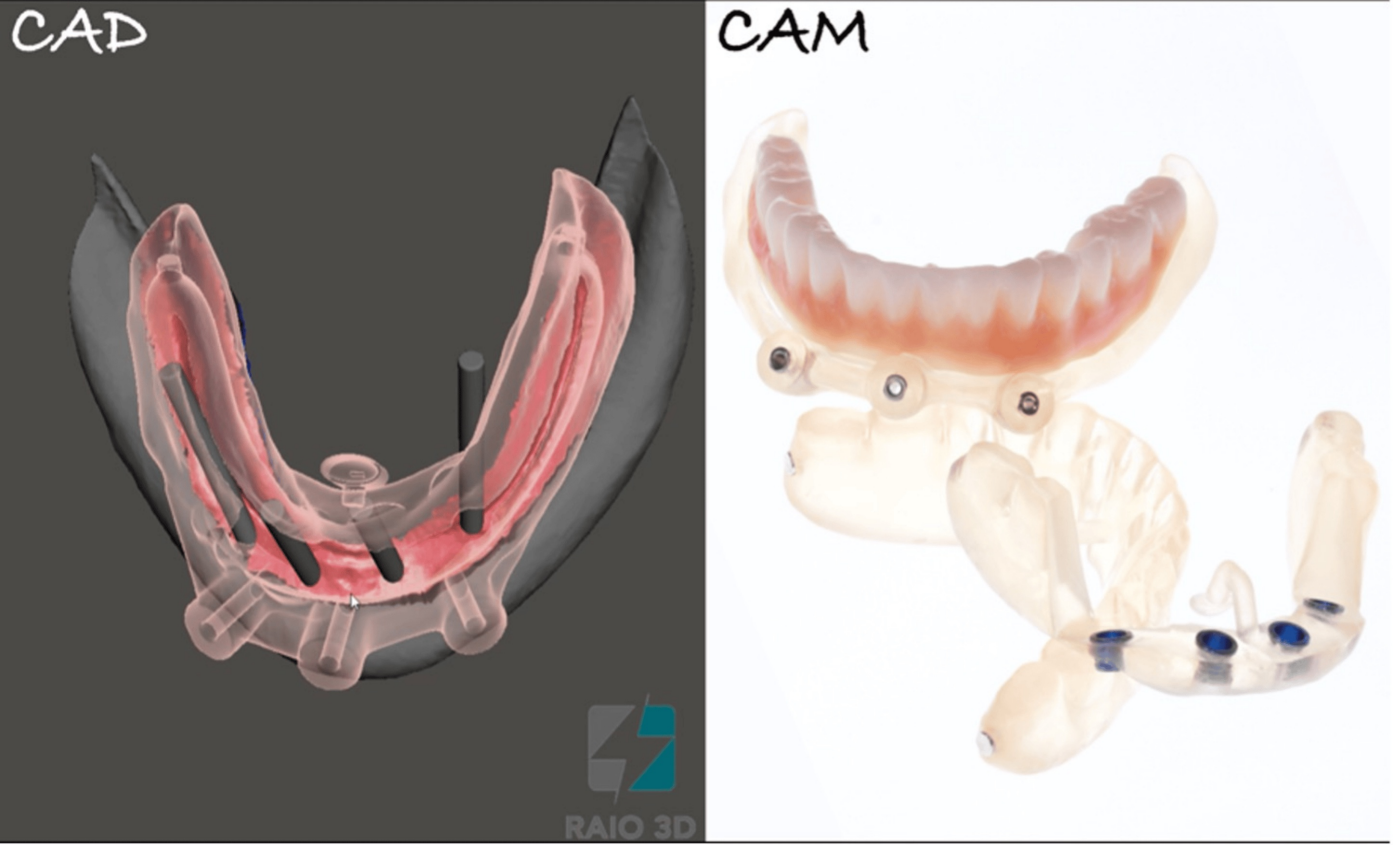
Electronic planning and printing (CAD/CAM) of the surgical guide and temporary prosthesis CAD/CAM: computer-aided design/computer-aided manufacturing

**Figure 3:**
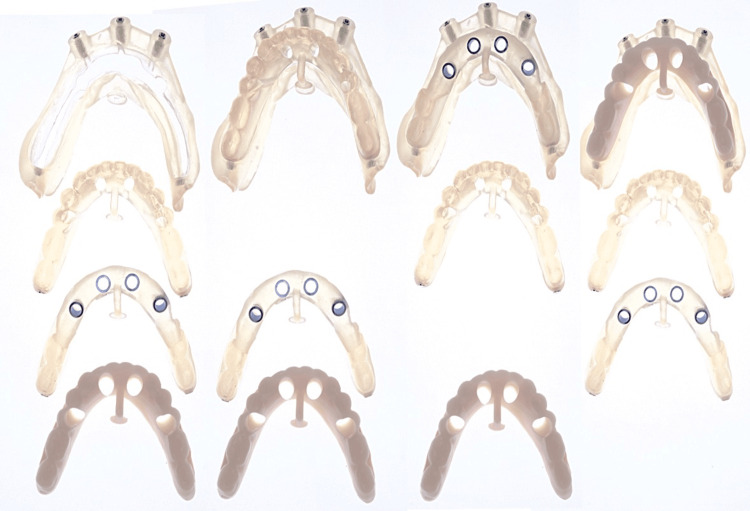
Sequence of prototype fittings using magnets* ^*^Dental implants (Maestro, Implacil - Osstem, São Paulo, Brazil), including two distal implants with a diameter

Digital scans of the existing prosthesis, combined with CBCT data (bone density D1 for the anterior implants and D2 for the posterior implants) featuring radiopaque markers, were used to create a virtual treatment plan with Blue Sky Plan software (Blue Sky Bio, Libertyville, IL). Implant positioning was digitally planned based on both anatomical and prosthetic considerations. The digital planning files were sent to Raio 3D Company (Porto Velho, RO, Brazil) and planned by Dr. Renata Tarnoschi Bordgnon for the fabrication of a custom three-part stackable surgical guide, Bio Guide (Makertech, São Paulo, Brazil), and a stereolithographically printed provisional prosthesis made from biocompatible resin (Bio Crown, Makertech, São Paulo, Brazil) (Figures 2, 3). The fit between the tomographic base and the custom guide was confirmed clinically, with no signs of misalignment or tissue impingement.

**Figure 4:**
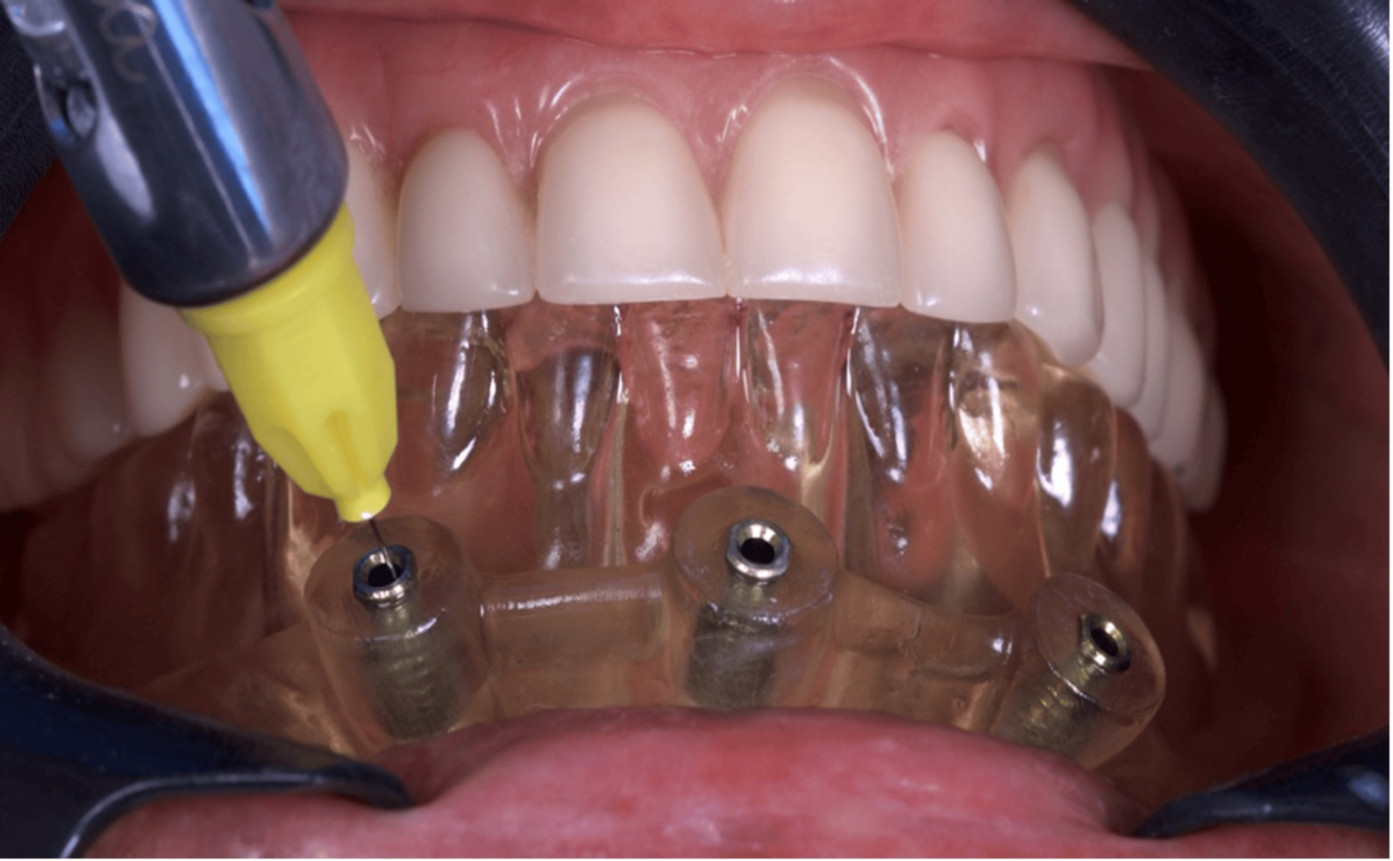
Anesthesia for guide fixation through milling and pin insertion

**Figure 5:**
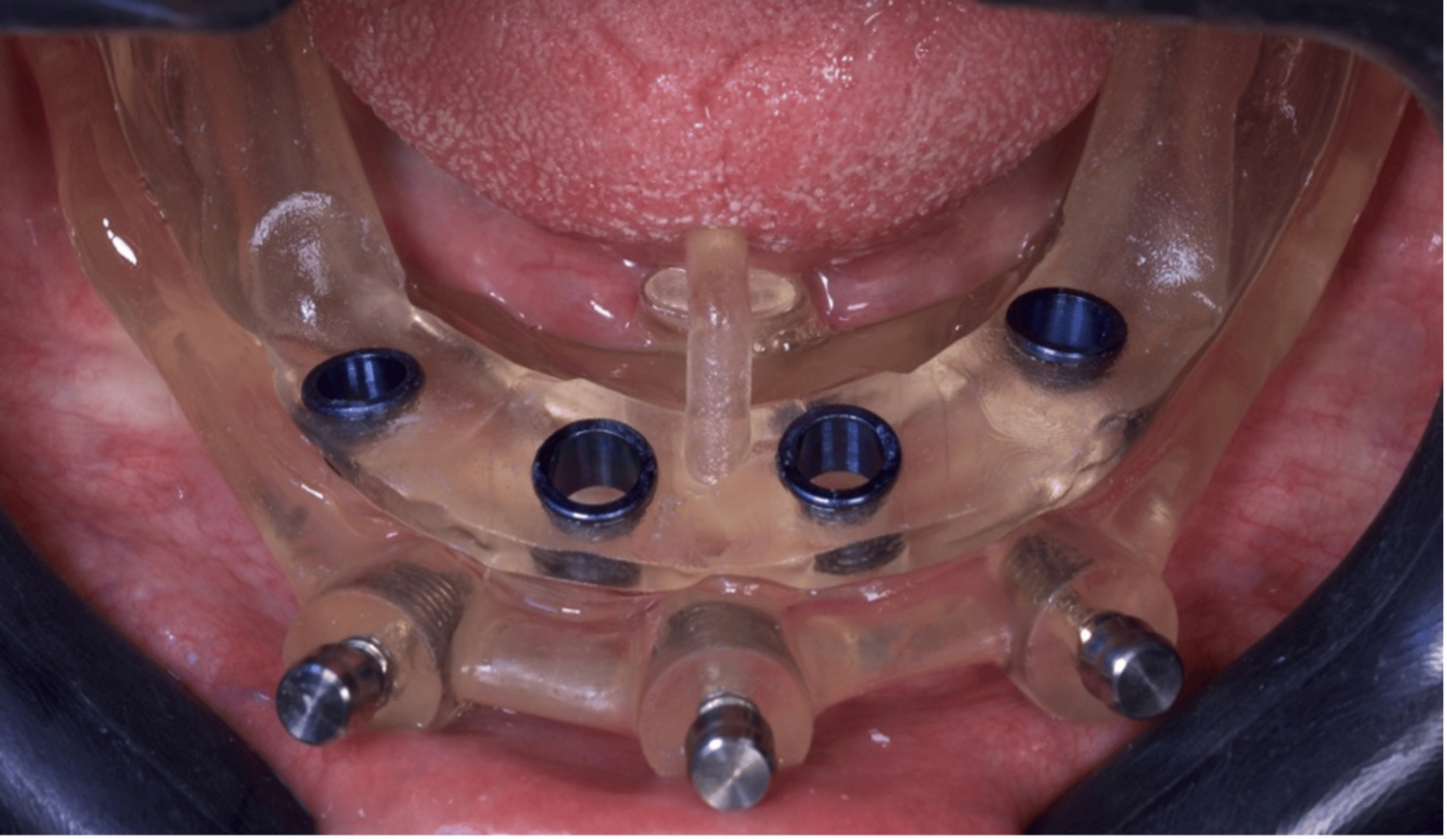
Occlusal view after removing the stabilization guide

**Figure 6:**
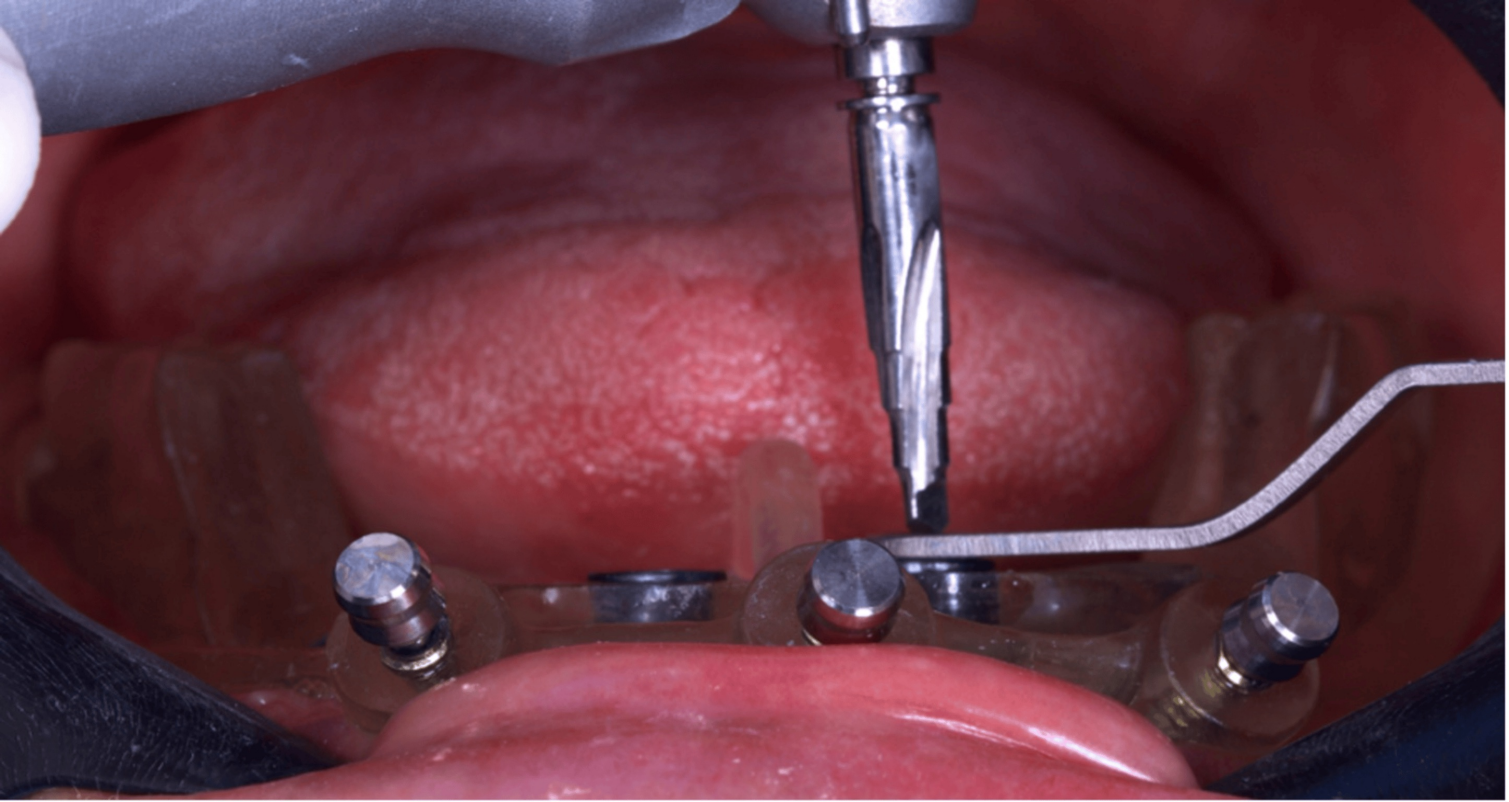
Fitting the bur through the surgical guide*, preparing the bone for the implant placement. ^*^Implaguide 3.5/4.0 mm ø; Implacil - Osstem, São Paulo, Brazil

**Figure 7:**
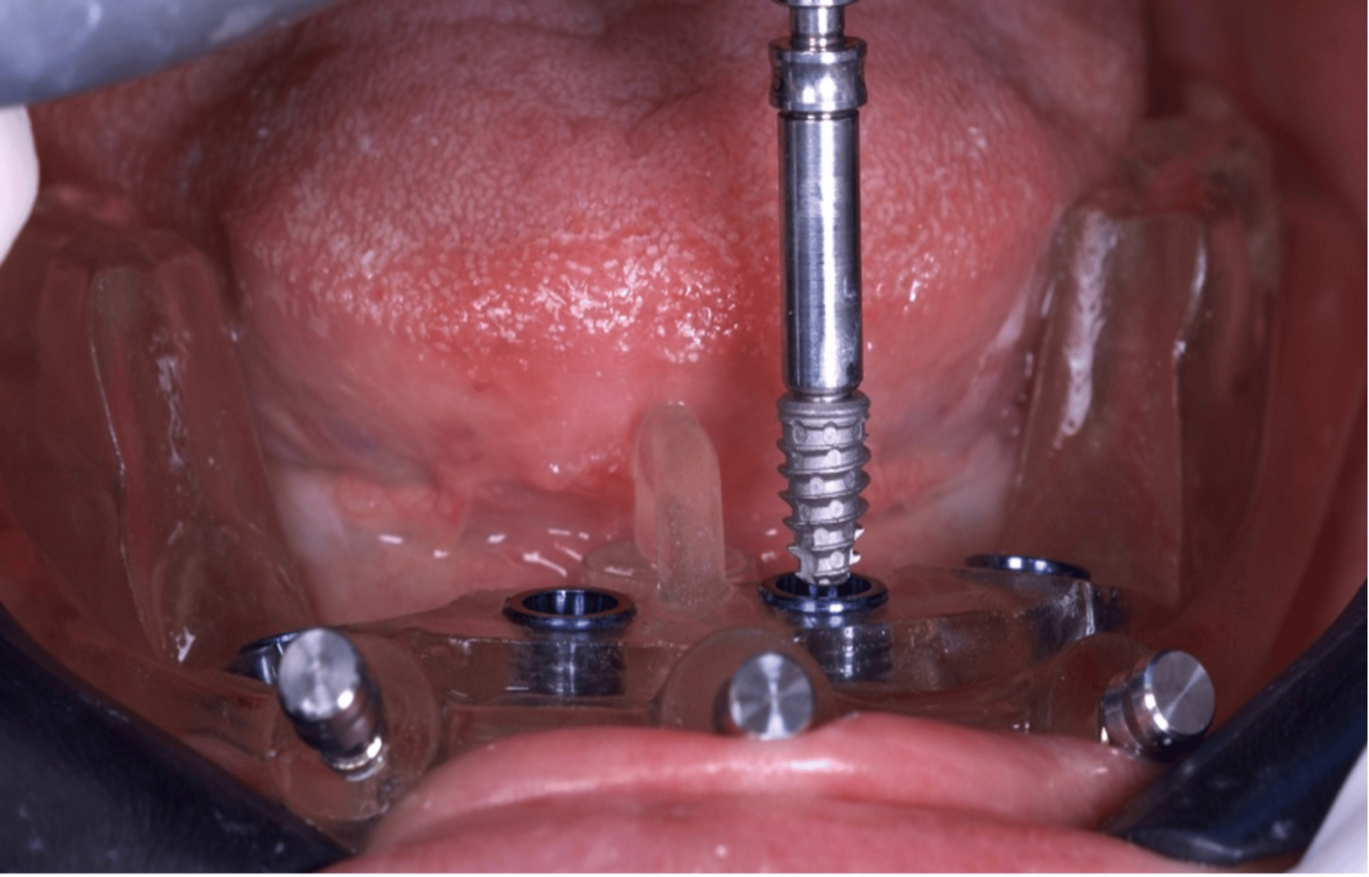
Implant placement is performed using the surgical guide previously created

**Figure 8:**
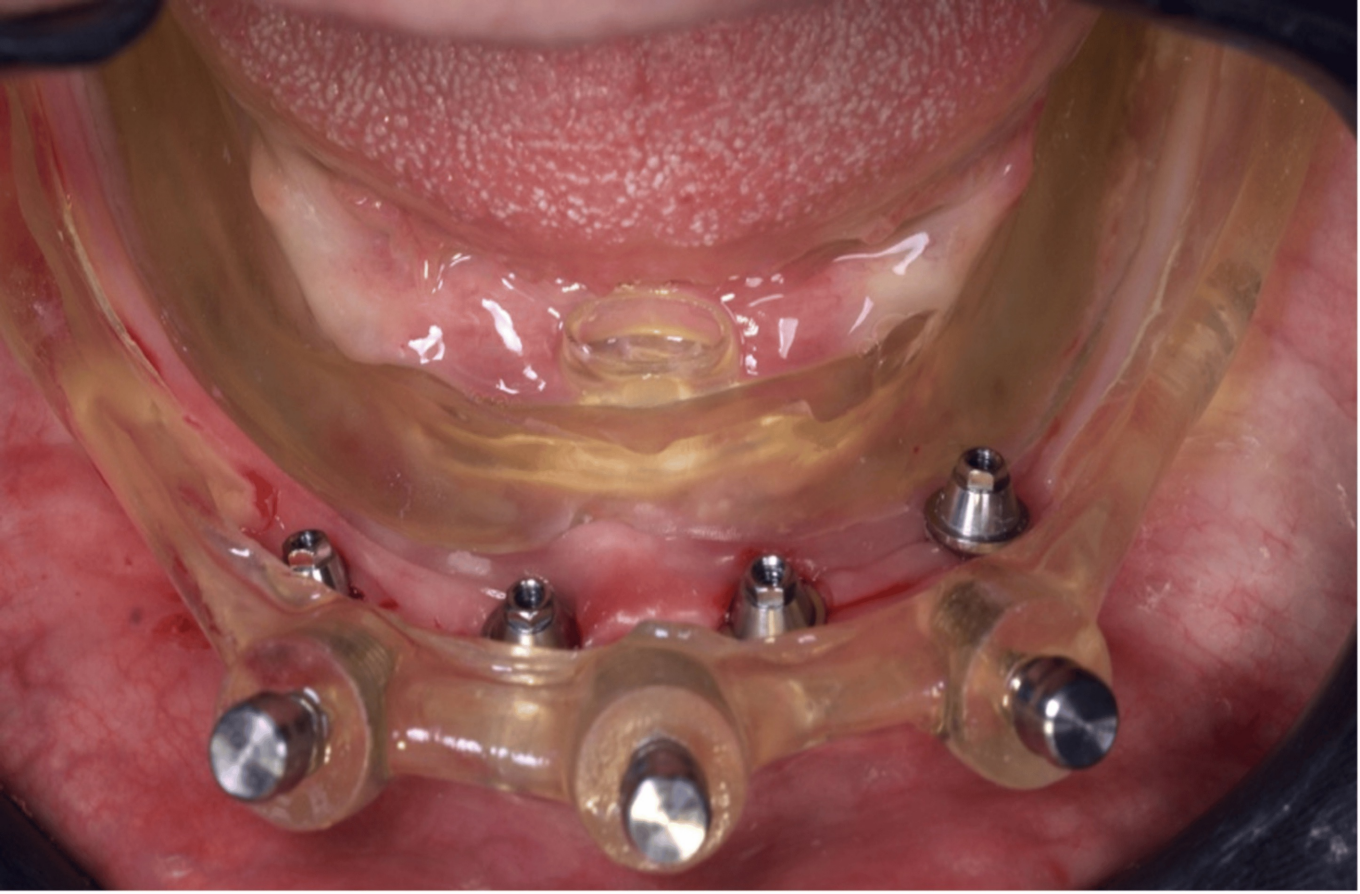
Removal of the milling guide and installation of the mini-pillars abutments* ^*^Mini-conico Implacil - Osstem

Guided implant surgery was performed under local anesthesia using 2% mepivacaine hydrochloride with epinephrine (1:100,000). The surgical guide was initially positioned and verified intraorally to check passivation and stable seating. Once the occlusal stabilization was achieved with the upper prosthesis, the occlusion guide was removed, and the milling guide was engaged to facilitate osteotomy preparation. Dental implants (Maestro, Implacil - Osstem, São Paulo, Brazil), including two distal implants with a diameter of 4.0 mm and a length of 11.5 mm and two central implants with a diameter of 4.0 mm and a length of 10 mm, were placed with primary stability exceeding 32 N·cm (between 40 and 50 N·cm), allowing immediate loading. The milling guide was then removed, followed by the installation of mini-pillar abutments (Mini-conicos, Implacil de Bortoli, São Paulo, Brazil) (Figures 4-8).

**Figure 9:**
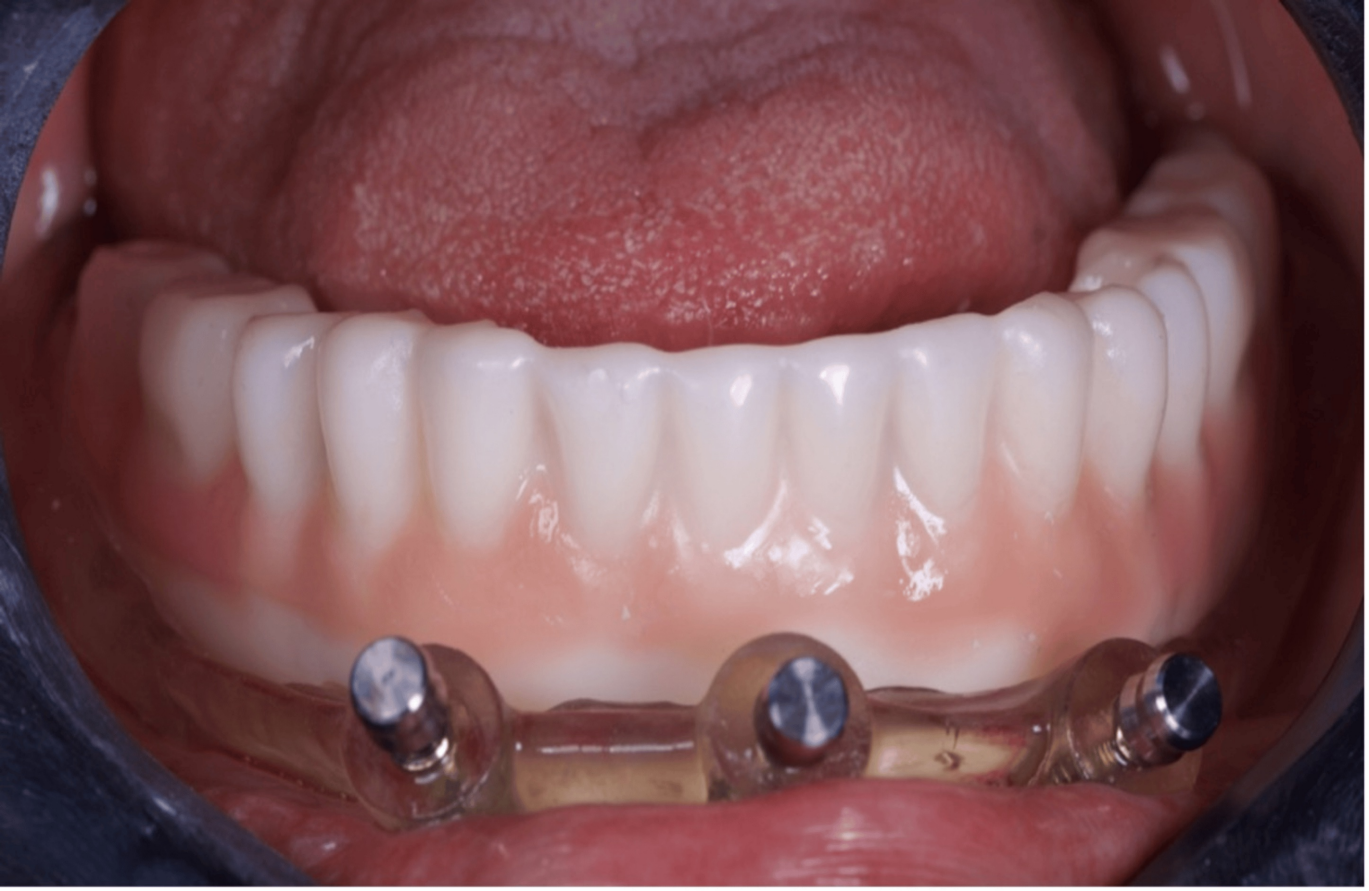
Fitting the temporary prosthesis into the base of the guide

**Figure 10:**
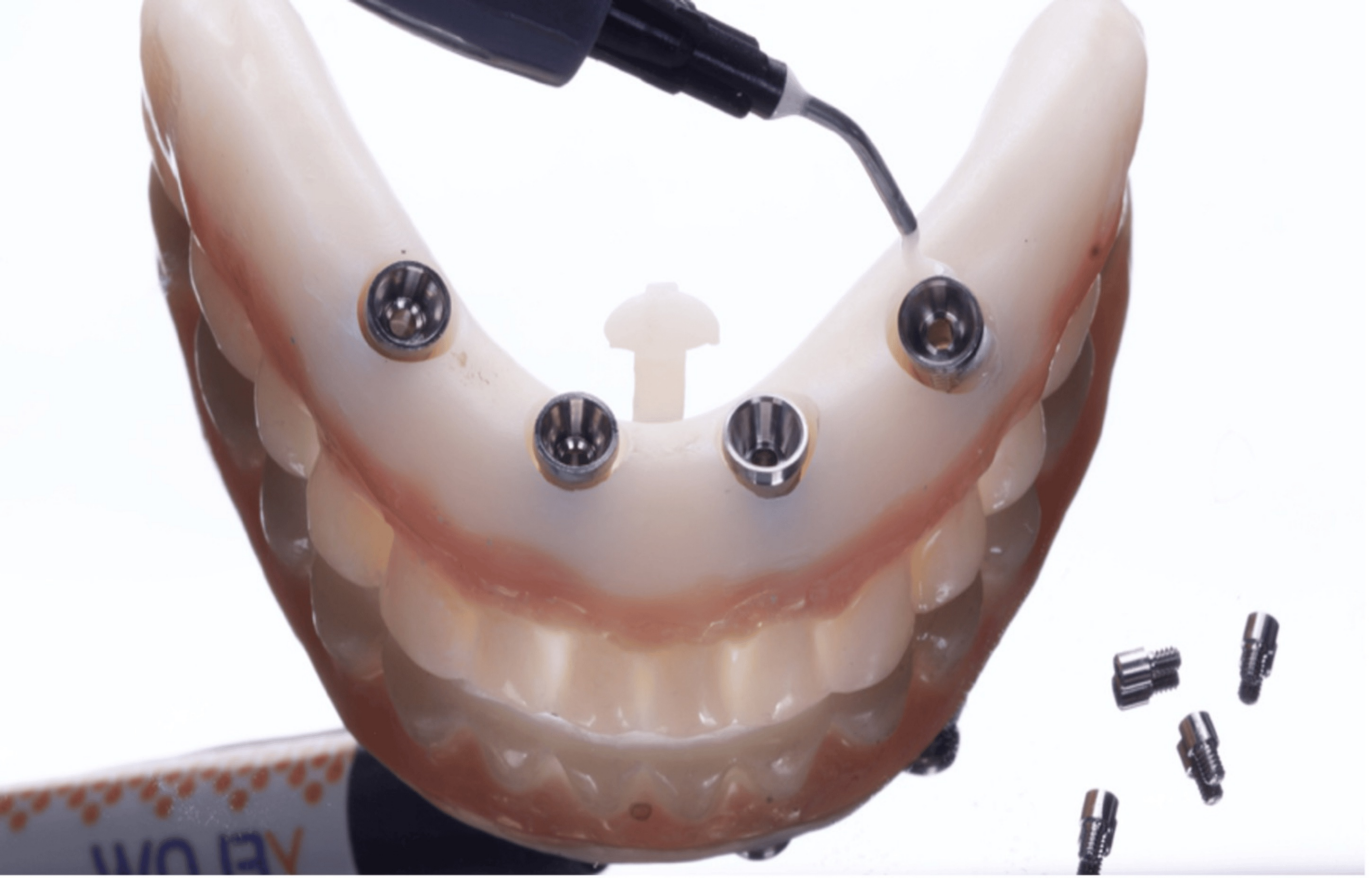
Capture of the prosthesis with flow resin and closure of spaces outside the mouth* ^*^Coifa for Mini-conics Implacil - Osstem

**Figure 11:**
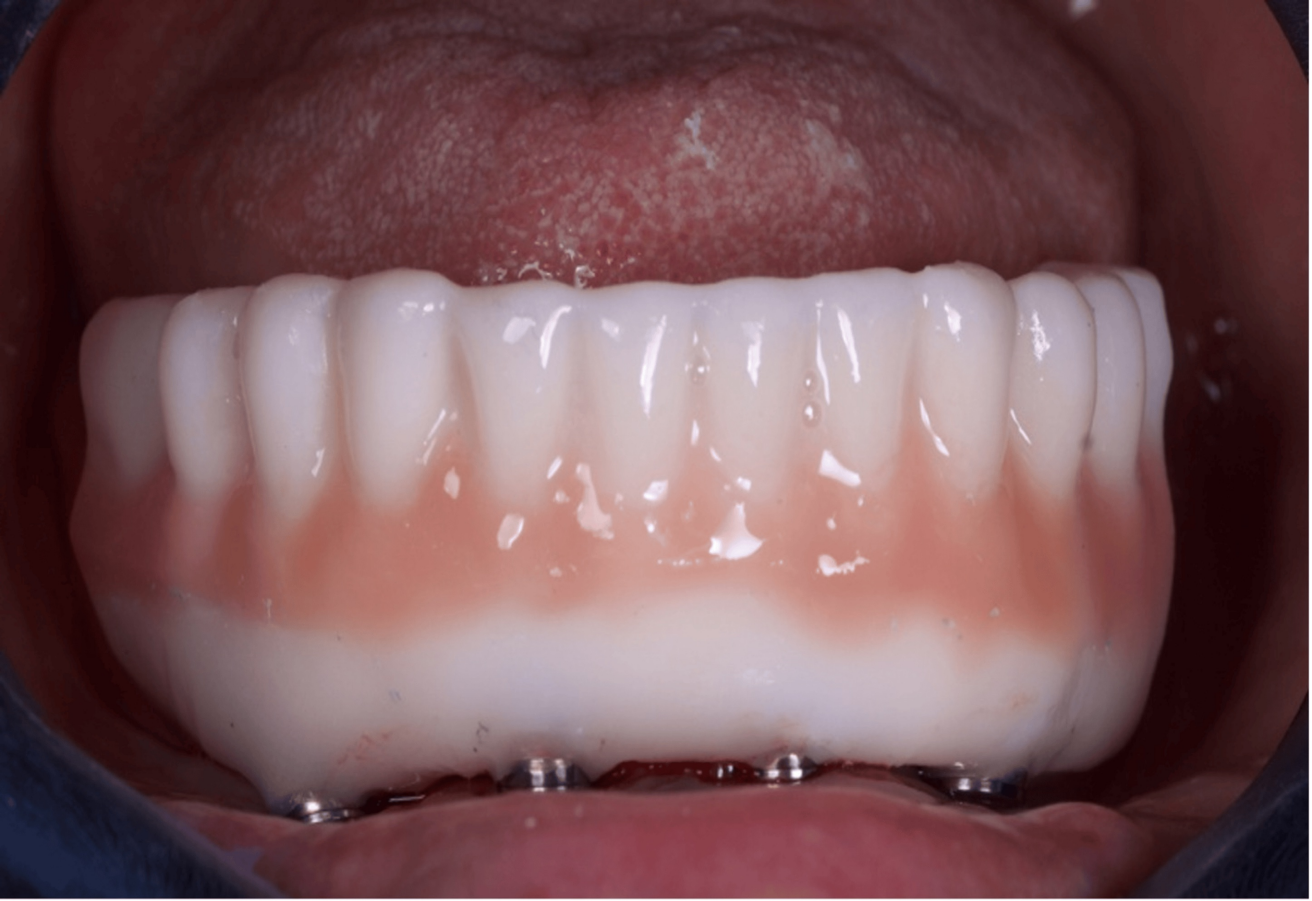
Installation of the temporary prosthesis for immediate loading

**Figure 12:**
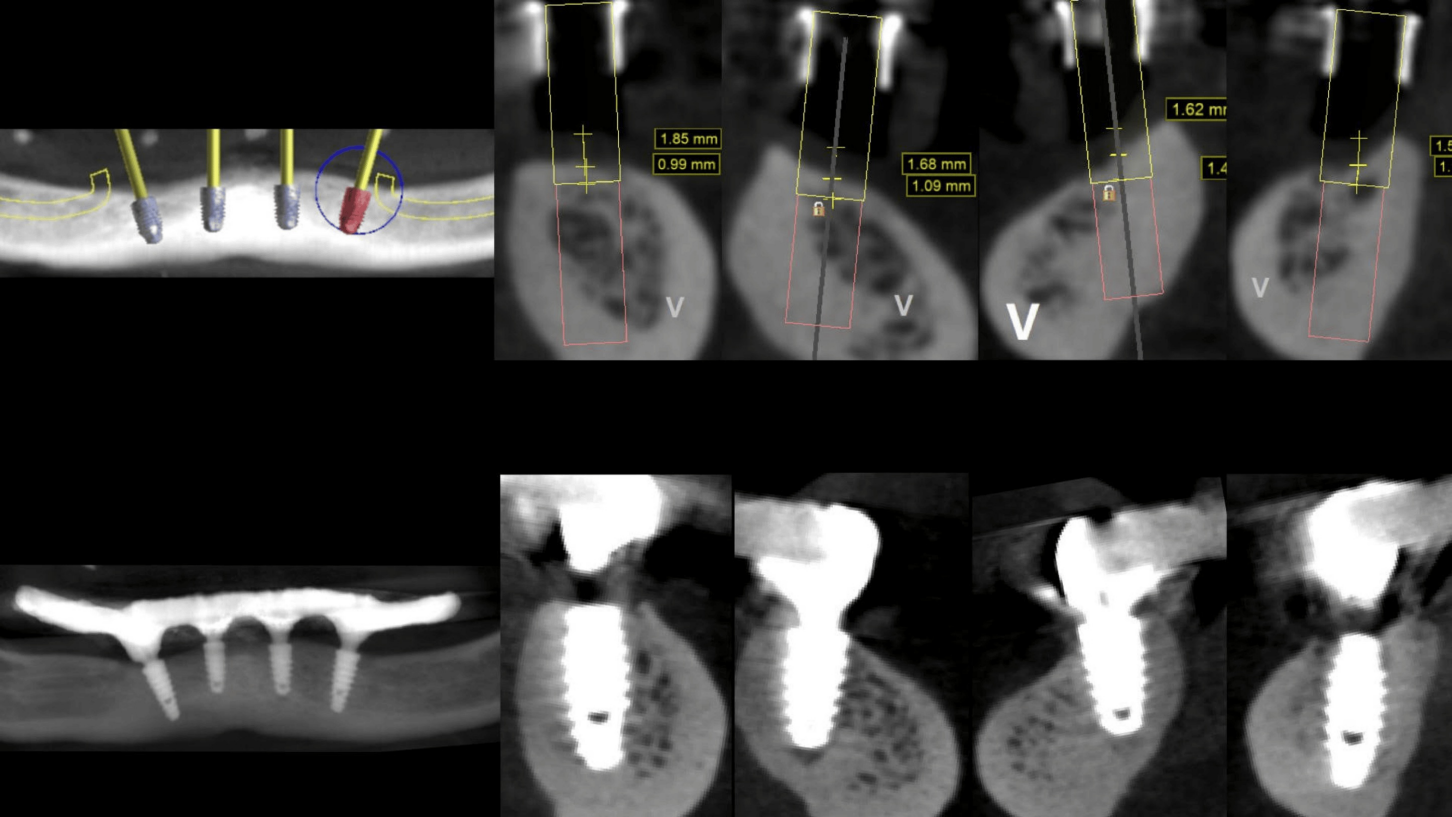
Initial software planning and the final panoramic result of implant placement with immediate provisional fixed rehabilitation Tomograph images show the results (right)

**Figure 13:**
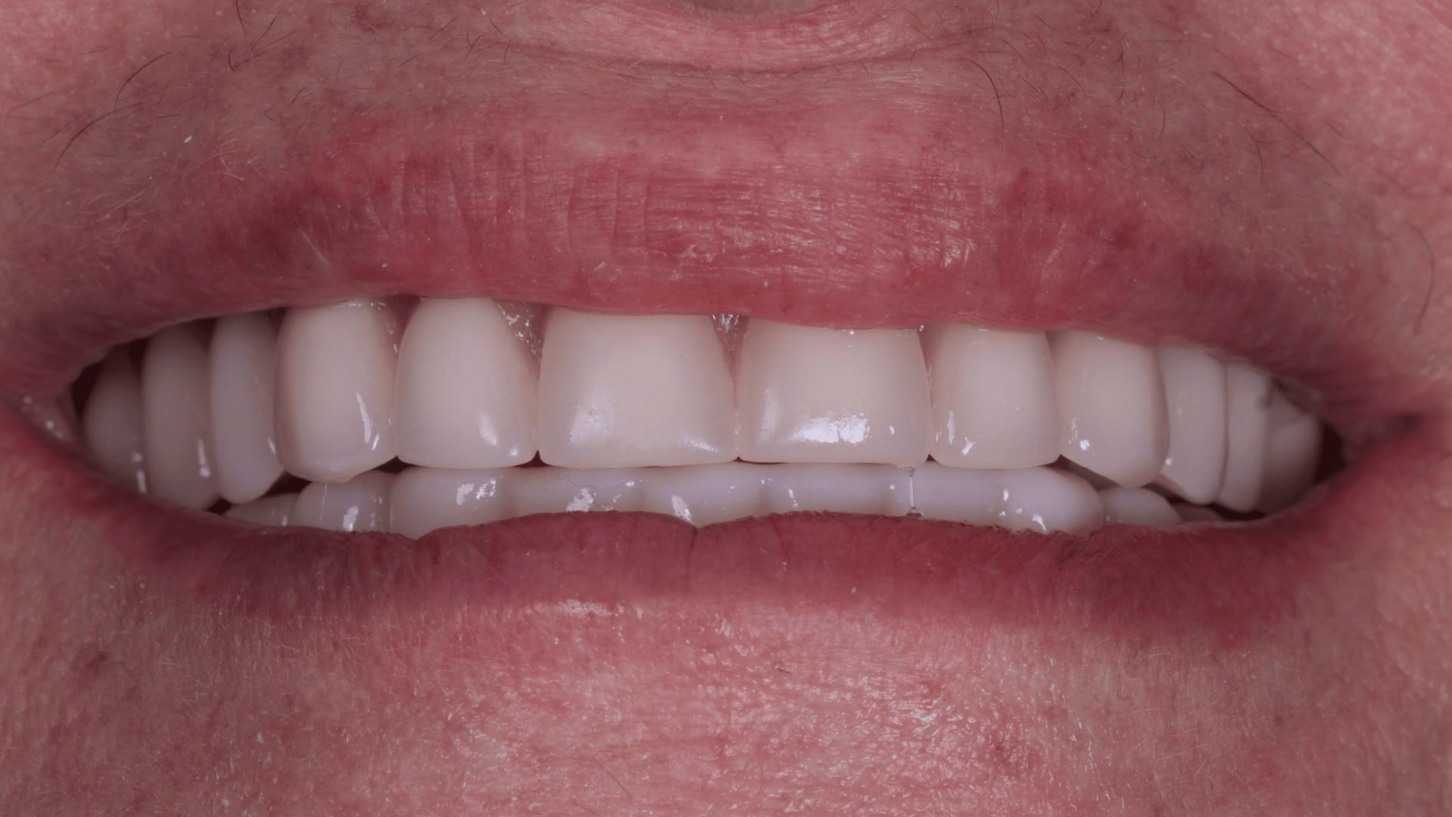
Final occlusion - frontal view

The provisional prosthesis was directly relined and secured using titanium abutments and flowable resin (Figures 9, 10). The installation of the temporary prosthesis for immediate loading was performed (Figure 11). The final panoramic radiograph and tomography image showing the result was obtained, revealing the implant placement with immediate provisional fixed rehabilitation (Figures 12, 13).

The total surgical and prosthetic procedure time, from anesthesia to the placement of the temporary prosthesis, was approximately two hours and 10 minutes. The immediate postoperative findings and adverse symptoms found were (1) mild to moderate pain or discomfort at surgical sites (controlled with medication), (2) swelling and edema of the mandibular mucosa and surrounding tissues, (3) minor bleeding within the first day after surgery, (4) low difficulty with speech or mastication during the adaptation phase, and (5) minor discomfort during denture use (first week).

After 24-48 hours post-surgery, the patient was reevaluated to observe the inflammatory burden, which was well-controlled. After seven days, the patient returned for suture removal and healing assessment. In the first month after surgical and prosthetic procedures, the soft tissue was checked and presented in a healthy condition, and the occlusion was adjusted.

## Discussion

The rapid advances in technology have led to significant changes in all fields of dentistry [18]. Implantology is no exception to these changes, and currently, specialized software is available to perform nearly all computer-guided surgical procedures [8], providing greater precision and foresight. In this technological era, it is valuable for surgeons to have all the support that technology can offer [7]. It is equally important for professionals to be prepared to utilize this technology fully, thereby reducing risks and procedure times for the benefit of the patient [8]. Digitization has had a profound impact on implantology, enabling the performance of treatments with greater precision, predictability, and reduced operating time. However, for this, the professional must invest more time in planning the case, have greater communication with the digital laboratory, be more aligned with the management of the different planning software, and print the surgical guides necessary for the case [8,18].

Total dentures, implant-supported overdentures, and fixed prosthesis-retaining implants (e.g., the All-on-4^®^ technique) represent three distinct approaches to the rehabilitation of edentulous patients, each with its own clinical implications, benefits, and drawbacks. This synthesis examines their comparative effectiveness in terms of patient satisfaction, functional outcomes, treatment costs, and biological impacts on bone. Regarding functional outcomes and patient satisfaction, total dentures often lead to functional challenges such as reduced bite force and decreased chewing efficiency. Studies indicate that complete denture wearers experience a substantial drop in bite force, up to 53% less than individuals with natural teeth or fixed prostheses [19]. In contrast, implant-supported overdentures and the fixed prosthesis-retaining implants significantly enhance masticatory function, with reduced chewing cycles noted for patients receiving fixed rehabilitations compared to those using conventional dentures [20,21]. The fixed approach, as used in the present case report, employs four strategically placed implants to support a fixed prosthesis, enabling functional outcomes comparable to those of fully dentate patients, and often yields higher patient-reported satisfaction scores compared to overdentures and complete dentures [22].

The impact of these rehabilitative strategies on bone health is critical. Patients using traditional complete dentures often experience significant bone resorption; for instance, resorption rates can reach around 5.2 mm compared to only 0.5 mm in those utilizing implant-supported overdentures over five years [23. The fixed method also offsets bone loss more effectively than complete dentures, resulting in limited marginal bone loss and preserving alveolar ridge integrity [24]. Notably, reports indicate that the cumulative success rates for implant-supported overdentures remain high, further supporting their role in maintaining bone health [25]. Otherwise, from a cost-effectiveness perspective, overdentures, particularly when compared to fixed complete dentures as those offered in the present case report, are generally found to provide a favorable balance of cost and benefit. A systematic review pointed out that although the initial financial outlay for implant-supported prostheses is higher, they can be more cost-effective over the long term due to lower rates of failure and the reduction in the need for adjustments or replacements seen with traditional dentures [21]. The All-on-4^®^ system has been highlighted as providing a cost-effective solution, considering its durability and the quality-of-life improvements it affords [26].

Regarding the biological and clinical complications, while both overdentures and fixed techniques offer substantial improvements in quality of life, attention must be paid to potential biological complications. The fixed hybrid prostheses are associated with particular biological and prosthetic challenges, such as complications linked to the prostheses themselves [27]. Overdenture patients, however, display a more favorable profile regarding inflammation and tissue health owing to the better distribution of forces and lesser trauma experienced by the supporting tissues [28]. Nonetheless, complications stemming from implant failure can occur, necessitating a thorough patient evaluation before rehabilitation [29].

The literature on the All-on-4^®^ rehabilitation technique emphasizes its efficacy and reliability as a treatment for edentulous individuals. This implant-supported approach involves placing four implants (two in the anterior region and two tilted implants in the posterior), thus providing a stable base for a full-arch prosthesis. Studies show that the All-on-4^®^ system yields a high implant survival rate of approximately 89.85% over an average of 5.9 years, making it a viable alternative to traditional dental rehabilitation methods, particularly for patients with severe bone loss [30]. The biomechanical advantages of this technique are further supported by research demonstrating that appropriately designed implants optimize stress distribution in the surrounding bone, which is crucial for long-term success and minimizing complications such as implant failure [31]. Researchers have employed various methodologies, including finite element analysis, to demonstrate that the angulation and position of implants significantly impact stress distribution, confirming that the All-on-4® technique can effectively reduce stress on bone structure compared to conventional implant placements [31].

Furthermore, with immediate loading protocols and a minimally invasive surgical approach, the All-on-4^®^ method also addresses patient needs for both functionality and esthetics promptly after surgery [32]. Thus, the All-on-4^®^ technique represents a robust, evidence-based choice for dental rehabilitation in edentulous patients. Even though immediate loading in the maxilla is well-documented in the literature, reports regarding osteotomy, implant installation, and rehabilitation - all of which are digitally assisted - are scarce, as is commonly observed in the mandibular arch, as seen in this case report. Then, the objective of this case report, which includes a digital protocol, was to provide a practical, predictable solution with greater precision, lower time consumption, and a better cost-benefit for the patient, improving the predictability of All-on-4® protocols [33,34].

Thus, for the development of the procedure in this case, a stackable guide was necessary. It was planned to transfer the virtual plan to the operating clinical field, intraorally, which consisted of 3 components: (1) carrier guide: it takes its support from the remaining dentition and has a foundation base attached to it by locks. Its function is to move the foundation base to its exact place in the patient’s mouth; (2) foundation base: fixed to bone during the entire time of surgery, utilizing fixation pins and has locks for its fixation with posterior drilling guides. It also serves as an osteotomy guide, and (3) a drilling guide, which contains the hole through which the implant drill will be guided.

The advantage of this stackable guide was to ensure an exact reproduction of what had been digitally planned, providing greater precision in the surgical steps. Additionally, it facilitates the perfect fit of the digitally designed printed prosthesis. For this reason, the positioning of the first carrier guide is crucial for the success of this surgical-rehabilitation protocol. Taking the time to ensure the perfect fit of this first guide is crucial. Any failure of this initial guide can impact the installation of the final prosthesis. This stackable guide solution is ideal for complex cases that require high levels of precision, adaptability, and flexibility. A comprehensive 3D digital design process was employed to create an efficient and reliable solution for complex procedures. Its ability to facilitate the accurate placement of surgical components and the seamless fit of the digitally printed prosthesis makes it an invaluable tool. Thus, as mentioned above, the correct positioning of the first guide is critical, as it lays the foundation for the success of the entire protocol. By prioritizing precision and investing time in the proper fit of this initial component, clinicians can significantly enhance outcomes, as seen in this case report. This digitally driven, adaptable solution represents a significant advancement in achieving predictable, efficient, and high-quality results in demanding clinical cases.

## Conclusions

This report demonstrates the effectiveness of a fully guided, computer-assisted surgical approach for immediate implant placement and provisional rehabilitation using a 3D-printed resin hybrid prosthesis. Digital planning and the use of a custom three-part stackable guide enabled the precise execution of multiple extractions, osteotomies, and implant placement, allowing for immediate functional loading in a single session of just over two hours. The outcome highlights the advantages of a digitally integrated workflow in complex full-arch rehabilitations, offering accuracy, efficiency, and patient-centered results aligned with modern implant dentistry standards. It should be noted that the provisional prosthesis may require replacement with a definitive prosthesis in the future to ensure long-term function, better esthetics, and durability.
